# Probabilistic Risk Assessment of Metals, Acrylamide and Ochratoxin A in Instant Coffee from Brazil, Colombia, Mexico and Peru

**DOI:** 10.3390/foods13050726

**Published:** 2024-02-27

**Authors:** Grobert A. Guadalupe, Dorila E. Grandez-Yoplac, Erick Arellanos, Eva Doménech

**Affiliations:** 1 Instituto de Investigación para el Desarrollo Sustentable de Ceja de Selva (INDES-CES), Universidad Nacional Toribio Rodríguez de Mendoza de Amazonas, 342 Higos Urco, Chachapoyas 01001, Peru; 2 Instituto de Investigación, Innovación y Desarrollo para el Sector Agrario y Agroindustrial de la Región Amazonas (IIDAA), Universidad Nacional Toribio Rodríguez de Mendoza de Amazonas, Chachapoyas 01001, Peru; yoplacesteffany@gmail.com; 3 Instituto de Investigación en Ingeniería Ambiental (INAM), Universidad Nacional Toribio Rodríguez de Mendoza de Amazonas, Higos Urco 342, Chachapoyas 01001, Peru; erick.arellanos@untrm.edu.pe; 4 Instituto Universitario de Ingeniería de Alimentos Food-UPV, Universitat Politècnica de València, Camino de Vera s/n, 46022 Valencia, Spain

**Keywords:** chemical analysis, risk characterisation, genotoxic and non-genotoxic effects, consumer health

## Abstract

This study analysed the probabilistic risk to consumers associated with the presence of iAs, Cd, Cr, Hg, Pb, acrylamide (AA) and ochratoxin A (OTA) in instant coffee from Brazil, Colombia, Mexico and Peru. The results found iAs to be the metal with the highest concentrations (3.50 × 10^−2^ to 6.00 × 10^−2^ mg/kg), closely followed by Pb (1.70 × 10^−2^ to 2.70 × 10^−2^ mg/kg) and Cr (5.00 × 10^−3^ to 1.00 × 10^−2^ mg/kg), although these differences were not significant between countries. Cd and Hg were not detected. Focusing on AA, the concentrations ranged from 1.77 × 10^−1^ mg/kg (Peru) to 4.77 × 10^−1^ mg/kg (Brazil), while OTA ranged from 1.32 × 10^−3^ (Peru) to 1.77 × 10^−3^ mg/kg (Brazil) with significant differences between countries in both cases. As regards risk, the hazard quotient and hazard index were less than 1, meaning that the consumption of instant coffee represents a low level of concern for non-genotoxic effects. The results of the combination of margin of exposure and probability of exceedance indicated that the non-genotoxic effects of Pb, AA and OTA pose no threat. However, the probability values of suffering cancer from iAs and AA (between 1 × 10^−6^ and 1 × 10^−4^) indicated a moderate risk and that management measures should be taken.

## 1. Introduction

Coffee has now become the second most popular hot beverage, appreciated for its stimulating effect and organoleptic characteristics. The International Coffee Organization (ICO) estimated that world coffee consumption increased by 4.2% to 175.6 million 60 kg bags in 2021/22, representing € 165 billion per year [1]. Instant coffee accounts for the second form of coffee exports and represents 8.2% of the world’s market share of the total coffee consumed [2]. It is obtained by removing water from the coffee extract by techniques such as spray-drying or freeze-drying. The dry powder thus obtained has a moisture content of between 2% and 4% [3,4,5,6,7]. Instant coffee is widely consumed in Eastern Europe (45%), Asia/Pacific (53%), and Australia (79%), and has become established in countries where tea was traditionally consumed, such as the United Kingdom, where 90% of the coffee consumed is instant [8,9], a fact that has led many producing countries, including Brazil, Colombia, Mexico and Peru, to increase their production of instant coffee in recent years [10].

Different chemical hazards produced when cultivating, processing and storing coffee can jeopardize the consumer’s health. These compounds include heavy metal(oid)s, acrylamide or mycotoxins, whose presence and potential impact on instant coffee are of growing scientific interest, especially in countries whose economic development depends directly on coffee exports [11]. The heavy metals of natural or anthropomorphic origin present in the environment enter the food chain, causing a wide range of toxic and mutagenic effects in the organism [12,13,14]. The toxicity of arsenic is partly related to its form, valency, solubility, rate of absorption and elimination from the organism, so that organic arsenic is rapidly absorbed and excreted, while inorganic arsenic (iAs) is considered highly toxic and carcinogenic. A cadmium (Cd) exposure in food higher than 4 × 10^−3^ mg/kg/day can cause renal dysfunction, cardiovascular disorders, cancer and other illnesses [15,16,17,18]. Chromium (Cr), an essential nutrient for humans, is involved in synthesising nucleic acids and the proper functioning of the immune system [19]. However, clinical research has found that acute prolonged exposure to Cr can cause genotoxicity [20]. Mercury (Hg) is transformed into methylmercury by microbial action, its most toxic organic form since it is soluble and accumulates in the adipose tissue of animals and humans, mainly affecting the central nervous system and crossing the blood–brain barrier and the placenta, which can cause alterations in the neuronal development of the foetus and young children. Also, above the permissible limits, lead (Pb) increases the risk of high blood pressure, cardiovascular problems and kidney damage [17,21,22]. Previous studies have shown that mineral content is influenced by soil characteristics, biochemical plant parameters, variety, and processing stages [14,23,24,25,26,27]. Another aspect of interest is the optimisation of detection and quantification in the analytical method, improving the sensitivity and precision of the results [28,29].

Acrylamide (AA) is a by-product of the Maillard reaction of asparagine with reducing sugars, such as glucose, galactose and fructose, at temperatures above 120 °C and peaking at around 170 °C [30,31,32,33]. AA is a genotoxic and neurotoxic compound, classified as level 2A since 1994 (probable human carcinogen) by the International Agency for Research on Cancer [34,35,36]. Coffee is one of the main contributors to the intake of this hazard, as the roasting and spray-drying stages play an essential role in forming AA [33,37]. To ensure consumer protection on food safety, the European Commission has set a benchmark level of 8.50 × 10^−4^ mg per kg of AA in instant coffee [38].

Ochratoxin A (OTA) is a secondary metabolite produced by filamentous fungi, mainly of the genera Aspergillus and Penicillium [3]. The main origin of this metabolite in coffee is related to manufacturing conditions, which include processing and storage activities that can favour fungal growth, such as the inherent hygroscopicity of coffee beans, international transport time or the climatic and geographical conditions of the growing area [39]. OTA contamination is now a public health problem as it has toxic effects on the kidneys and liver and is also associated with possible adverse effects on the nervous, haematological and immune systems [40]. The International Agency for Research on Cancer has classified it as a class 2B carcinogen [41,42] and the European regulations have established a maximum OTA of 5 × 10^−3^ mg/kg in instant coffee [43].

The risk to the consumer can be determined by performing a risk assessment. This component of risk analysis is defined as a systematic process of identifying, analysing and characterising the risk to consumers’ health over a given period [44,45]. In this analysis, a probabilistic approach is essential to take into account both the variability of the input data (such as consumption, concentration of chemical contaminants, weight differences between population groups or digestibility) and also to reduce the uncertainty of the calculated risk [46,47,48,49]. In this approach, the input data are represented by a distribution, which must be combined to estimate the range and probability of a hazard, exposure or risk rather than a single-point estimate. This process is usually done by Monte Carlo simulation, resulting in a frequency distribution that provides not only the extreme values but also the most likely outcome [50]. On the other hand, humans are simultaneously exposed to many chemicals on a daily basis, so that their effect on health must be studied by considering their combined action, i.e., their additive, synergistic or antagonistic effect [51,52]. The aim of the present study was thus to assess the risk to consumers due to the presence of chemical contaminants generated in the field, such as metals (iAs, Cd, Cr, Hg and Pb) or due to storage and processing conditions (AA and OTA) in instant coffee from Brazil, Colombia, Mexico and Peru. The non-genotoxic effects were studied by calculating the hazard quotient and the hazard index (for the combined effect of Cd, Cr, Hg and AA) and the MOE-POE combination (of Pb, AA and OTA), and the genotoxic effects through calculating a MOE-POE combination (of iAs, AA and OTA) and the cancer risk (of iAs, Pb and AA), all under a probabilistic approach. This information could help manufacturers and health authorities to make informed risk decisions.

## 2. Materials and Methods

### 2.1. Study Material

A total of 180 instant coffee samples from Brazil (Sao Paulo and Belo Horizonte), Colombia (Bogota and Barranquilla), Mexico (Mexico City) and Peru (Lima and Chachapoyas) were purchased in retail outlets between July and September 2022. The samples were kept in the original containers (glass and bioriented polypropylene bags) and stored at room temperature until analysis. All the samples were analysed in triplicate.

### 2.2. Chemical Analysis

The chemical hazards studied in instant coffee, metals (iAs, Cd, Cr, Hg and Pb), AA and OTA were analysed at the Soil and Water Research Laboratory (LABISAG) of the Universidad Nacional Toribio Rodríguez de Mendoza de Amazonas in Peru.

#### 2.2.1. Heavy Metal(oid)s

The concentration of metals was determined following the method described by Leiva-Tafur et al. (2022) and Guadalupe et al. (2023) [24,53].

The acid digestion technique was used for sample preparation. One gram of the sample was weighed and placed in crucibles inside the muffle at a temperature of 450 °C for 6 h. The calcined sample was moistened with 3 drops of distilled water and 1 mL of concentrated HCl, leaving it to stand for 1 h. Subsequently, 1 mL of distilled water and 1 mL of concentrated HCl were added and left to stand for 1 h. Then, the solutions of the samples by filtration were transferred to a 25 mL flask and it was gauged with distilled water to take the volume to 25 mL for the analysis.

Total metals were determined by microwave plasma atomic emission plasma spectroscopy (ICP-AES) (Agilent 4100 MP-AES, Agilent Technologies, Santa Clara, CA, USA) equipped with a standard torch, an Inert One Neb nebulizer and a dual-pass glass cyclonic spray chamber (Agilent Technologies) [54].

Inorganic arsenic was determined by ion chromatography (IC) combined with inductively coupled plasma atomic emission spectroscopy [55]. Ultrapure water (4.1 mL) and concentrated HCl (18.4 mL) were added to the samples (0.5 g). The mixture was left overnight. The sample and extraction solvent were separated using a centrifuge and then the solution was filtered. HBr (2 mL) and 1.5% (*w*/*v*) hydrazine sulphate (1 mL) were added. The inorganic arsenic was extracted into 30 mL of chloroform, and then extracted into 20 mL of 1 M HCl. The solvent was removed by a rotary evaporator to about 1 mL. The evaporation flask was washed with ultrapure water. The solution was transferred to a 50 mL Falcon tube and the final volume was brought to 20 mL. 

Detection (LOD) and quantification (LOQ) limits were calculated as the minimum detectable amount of analyte with a signal-to-noise ratio of 3:1 and 10:1, respectively [56], and are expressed as concentration (mg/kg). Detection wavelengths of 228.802 nm, 405.781 nm, 193.695 nm, 425.433 nm and 253.652 nm were selected to quantify iAs, Cd, Cr, Hg and Pb, respectively.

The equipment was calibrated using standard solutions of each element in different concentrations, prepared from a standard solution of 1.00 × 10 mg/kg. The analytical quality of detection and quantification of iAs, Cd, Cr, Hg and Pb was controlled by measuring blind and double samples. The parameters were validated from ten replicates, for iAs, Cd, Cr, Hg and Pb, the limit of quantification (LOQ) was 1.00 × 10^−2^, 1.50 × 10^−2^, 1.00 × 10^−2^, 1.50 × 10^−2^ and 1. 50 × 10^−2^ mg/kg, the limit of detection (LOD) was 3.00 × 10^−3^, 5.00 × 10^−3^, 3.00 × 10^−3^, 5.00 × 10^−3^ and 5.00 × 10^−3^ mg/kg, and the linearity (R^2^) was 9.96 × 10^−1^, 9.99 × 10^−1^, 9.99 × 10^−1^, 9.99 × 10^−1^, 9.98 × 10^−1^ and 9.99 × 10^−1^, respectively. The laboratory is accredited as conforming to the standard [57], granted by the National Institute of Quality (INACAL) of Peru.

#### 2.2.2. Acrylamide

The AA was determined following the method described by Mesías and Morales (2016) [58]. The analysis was carried out on a high-performance liquid chromatograph (U-HPLC) (Agilent 1290 Infinity II, Santa Clara, CA, USA), coupled to an Agilent Triple Quadrupole MS detector (Agilent Technologies, Santa Clara, CA, USA) with positive electrospray ionization. The sample was separated on a Hypercarb column (50 × 2.1 mm, 5 µm; Thermo Scientific, Bremen, Germany) at 30 °C with formic acid in water eluent (2.00 × 10^−1^ mL/ 1.00 × 10^2^ mL) at a flow rate of 4.00 × 10^−1^ mL/min. The linearity (R^2^) was 9.997 × 10^−1^, the LOD was set at 5.00 × 10^−3^ mg/Kg and the LOQ at 1.00 × 10^−2^ mg/Kg.

#### 2.2.3. Ochratoxin A

OTA was quantified following the methodology described by Vecchio et al. (2012) [59]. Determinations were performed by ultra-high performance liquid chromatography (U-HPLC) (Agilent 1290 Infinity II, Santa Clara, CA, USA), equipped with a 20 μL loop and connected to a spectrofluorometer detector, Perkin Elmer Fluorescence Detector Series 200. The excitation and emission wavelengths were 330 and 460 nm. Chromatography was carried out isocratically using 4 mM sodium acetate/acetic acid (19:1): acetonitrile (60:40) as the mobile phase at a 1.0 mL/min flow rate. The working standard solution and sample volumes of 20 μL were injected in triplicate. The parameters were validated by six replicates. The LOD was 1.60 × 10^−5^ mg/kg, the LOQ was 4.80 × 10^−5^ mg/kg and the linearity coefficient (R^2^) was 9.997 × 10^−1^. The retention time was 9.09 ± 0.08 min.

### 2.3. Risk Characterisation

This section gives the equations and parameters involved in calculating the risk characterisation of the hazards studied, Table 1.

Risk characterisation was performed by a probabilistic approach; considering the random uncertainty of the input data and the AA and OTA concentrations of the samples studied, the consumption of instant coffee and the consumers’ weight were fitted to a probability density function (pdf) on @Risk version 8 software (Palisade, Newfield, NY, USA). The same program was also used to quantify risk by different metrics, by simulation with a standard Monte Carlo method and hypercube Latin sampling in 10 repetitions of 100,000 iterations, obtaining a pdf for each of the results. An HI of less than one was considered “acceptable” when interpreting the results, as the exposure to the hazard does not exceed the reference value, so that consumers are unlikely to suffer adverse health effects [60,61].

In interpreting the MOE, it is assumed that a value above 10,000 is of low concern from a public health point of view. For MOE values below this limit, the combination with the POE metric provides valuable information for making risk decisions. The POE is thus a measure of the probability that the change in the population’s response exceeds the predefined benchmark response. It could also be interpreted as the fraction of the total population exposed to increased risk. The POE ranges from zero to one, in which zero means that exposure is always lower than the reference value, and the risk can be considered negligible, while a POE value of one indicates that the reference value is always exceeded and must be considered as not tolerable. The POE metric can thus be used as a measure of the level of concern to complement the MOE [62]. With regard to the probability of developing cancer, values below 1 × 10^−6^ are not considered to be a health risk; between 1 × 10^−6^ and 1 × 10^−4^ the risk is acceptable, and above 1 × 10^−4^, the risk is high and indicates potential harm to consumers [63].

**Table 1:** Parameters and equations of interest for risk characterisation of chemical hazards (x) and country (i).

Parameter	Description	Value	Units	Source
HQxi	Hazard quotient	EDIxi/RVx		ATSDR, 2022
HIi	Hazard index per country	$\sum_{n=1}^{x}{HQ}_{n}$		ATSDR, 2022
EDIxi	Estimated daily intake	Cxi · IR/Bw	mg/kgBw/day	EFSA, 2013
Cxi	Concentration	Table 2	mg/kg	This study
IR	Ingestion rate	Exponential (5%; 6.82 × 10^−4^; 95%; 1.2 × 10^−2^)	kg/day	EFSA, 2023
Bw	Body weight	Gamma (2%; 54; 50%; 75; 98%; 110)	kgBw	CTCF, 2012
RVx	Reference Value	RfD * (Cr): 3 × 10^−4^	mg/kgBw/day	EPA, 2022 [64]
		RfD (Cd): 1 × 10^−3^	mg/kgBw/day	EPA, 2022 [64]
		RfD (Hg): 1 × 10^−4^	mg/kgBw/day	EPA, 2022 [64]
		RfD (AA): 2 × 10^−3^	mg/kgBw/day	EPA, 2022 [64]
MOExi	Margin of exposure	BMDL_%X_/EDIxi		EFSA, 2005
BMDL_%X_	Benchmark dose	BMDL01(iAs): Uniform (3 × 10^−4^; 8 × 10^−3^) (Carcinogenic)	mg/kgBw/day	EFSA, 2021
		BMDL01 (Pb): 1.5 × 10^−3^ (Cardiovascular)	mg/kgBw/day	EFSA, 2010 [65]
		BMDL10 (Pb): 6.3 × 10^−4^ (Nephrotoxic)	mg/kgBw/day	EFSA, 2010 [65]
		BMDL10 (AA): 1.7 × 10^−1^ (Carcinogenic)	mg/kgBw/day	EFSA, 2015 [66]
		BMDL10 (AA): 4.3 × 10^−1^ (Neurotoxic)	mg/kgBw/day	EFSA, 2015 [66]
		BMDL10 (OTA): 1.45 × 10^−2^ (Carcinogenic)	mg/kgBw/day	EFSA, 2020
		BMDL10 (OTA): 4.73 × 10^−3^ (Nephrotoxic)	mg/kgBw/day	EFSA, 2020
POExi	Probability of exceedance	$Pr\left({EDI}_{xi}>{BMDL}_{x}\right)=\int_{{BMDL}_{\%x}}^{\infty }f(E)dE$		Domenech & Martorell, 2021 [62]
CRxi	Cancer risk	EDIxi · SFx		ATSDR, 2022
SFx	Slope factor	SF (iAs): 1.5	(mg/kgBw/day)^−1^	EPA, 2022 [64]
		SF(Pb): 8.5 × 10^−3^	(mg/kgBw/day)^−1^	EPA, 2022 [64]
		SF(AA): 5 × 10^−1^	(mg/kgBw/day)^−1^	EPA, 2022 [64]

* The reference value used was the reference dose (RfD).

### 2.4. Statistical Analysis 

Multivariate ANOVA and Tukey’s mean comparison test were performed on SPSS V.25 statistical software to find any differences between the groups. A *p*-value of less than 0.05 was considered statistically significant, while the F-ratio (variability due to the considered effect divided by the residual variance) was obtained to analyse the factor’s effect on a variable.

## 3. Results and Discussion

### 3.1. Hazard Concentration

The results showed that Cd and Hg values were below the LOD, while the concentrations of iAs, Cr, Pb, AA and OTA were above it in all the countries studied. Table 2 gives the mean, standard deviation, minimum and maximum concentration of quantified hazards, the multifactor analysis of variance (ANOVA), F-ratio and significant differences for the factor: country of origin. Figure S1 shows the concentrations of iAs, Cd, Cr, Pb, AA and OTA found by other authors and those obtained in the present study.

The mean levels of iAs in the four countries studied ranged from 4.68 × 10^−2^ mg/kg in Colombia to 5.16 × 10^−2^ mg/kg in Peru. These values are higher than those obtained in a study carried out in Denmark by Pedersen et al. (1994) [67], in which the values ranged from 7.00 × 10^−4^ to 7.00 × 10^−3^ mg/kg. Values below the LOD (<1.00 × 10^−2^ mg/kg) were obtained by Dos Santos and De Oliveira in instant coffee from Brazil [25]. These different iAs concentrations in instant coffee can be related to very different variables such as the crop production area, the manufacturing process, the technique used to obtain instant coffee, the system of analysis, etc. [24,27,68].

The mean Cr values were of the order of 6.00 × 10^−3^ mg/kg in all the countries, being slightly higher in Peru (8.00 × 10^−3^ mg/kg). These values were higher by one order of magnitude than those obtained by Suseela et al. (2001) [69] in India (from 7.00 × 10^−4^ to 8.00 × 10^−4^ mg/kg). However, they were lower by two orders of magnitude to those found in the work by Dos Santos and De Oliveira [70] in samples from Brazil (5.20 × 10^−1^ mg/kg), Grembecka et al., (2007) [71] and Szymczycha-Madeja et al., (2015) [72] in instant coffee marketed in Poland (3.00 × 10^−1^ and 4.87 × 10^−1^ mg/kg, respectively), Pedersen et al., (1994) [67] in Denmark (2.30 × 10^−1^ mg/kg), Zaidi et al., (2005) [73] in Pakistan (1.07 × 10^−1^ mg/kg) and Oliveira et al., (2012) [74] in Portugal (5.00 × 10^−2^ mg/kg).

Our results on Pb contamination of instant coffee ranged from a minimum value of 1.70 × 10^−2^ mg/kg, obtained in Colombia, to a maximum of 2.70 × 10^−2^ mg/kg in Brazil. These values are in agreement with previous studies conducted in Poland by Winiarska-Mieczan et al. (2022) (8.26 × 10^−2^ mg/kg) [75] and in India by Suseela et al. (2001) (2.10 × 10^−2^ to 2.30 × 10^−1^ mg/kg) [69]. Concentrations notably lower or below the LOD were reported by Pedersen et al. (1994) (LOD—6.00 × 10^−4^ mg/kg) [67], Dos Santos and De Oliveira (2001) (<LOD: 1 mg/kg) [70], Grembecka et al. (2007) (<LOD: 1.00 × 10^−1^ mg/kg) [71], Szymczycha-Madeja et al. (2015) (<LOD: 2.70 × 10^−1^ mg/kg) [72]. The differences we found in iAs, Cr and Pb in the individual countries were not significant.

We also found a Cd of <LOD, in agreement with that obtained by Dos Santos and De Oliveira (2001) [70] in Brazil and slightly lower than those obtained by Pedersen et al. (1993) [67] in Denmark (2.50 × 10^−3^ mg/kg), Suseela et al. (2001) [69] in India (1.00 × 10^−3^ to 2.90 × 10^−2^ mg/kg) and Winiarska-Mieczan et al. (2022) (9.50 × 10^−2^ mg/kg) [75] and Grembecka et al. (2007) (1 × 10^−1^ mg/kg) [71] in Poland.

The findings for AA showed that the mean values ranged from 1.77 × 10^−1^ mg/kg in Peru to 4.77 × 10^−1^ mg/kg in Brazil. In relation to the maximum value, Brazil obtained the highest value, with 6.2 × 10^−1^ mg/kg. This result indicates that all the samples were below the limit of 8.5 × 10^−1^ mg/kg established by the EU (Regulation (EU) 2017/2158) [38]. On the other hand, the differences in AA content between the countries were significant (*p* < 0.05). Previous studies found that these differences can be attributed to many factors such as the type of coffee bean (Robusta and Arabica), the manufacturing process used (elimination of asparagine or its conversion into aspartic acid, which slows down the Maillard reaction), or the storage conditions (which can favour the formation of acrylamide in coffee) [30,76]. These results agree with numerous authors such as Granby and Fagt (2004) [77], Şenyuva and Gökmen (2005) [78], Baron Cortes et al. (2021) [79], Andrzejewski et al. (2004) [80], Loaëc et al. (2014) [81], Arisseto et al. (2007) [82], Surma et al. (2017) [83], Gonzalez et al. (2022) [84], and Lee et al. (2020) [85], whose values were from 1.07 × 10^−1^ to 7.1 × 10^−1^ mg/kg. Values in the order of 1 × 10^−2^ mg/kg were obtained in studies by Claeys et al. (2016) [86] and Health Canada (2012) [87]. A monitoring study carried out by EFSA (2015) showed that the mean AA values tested in instant European coffee were 7.16 × 10^−1^ mg/kg and 1.12 mg/kg at 95th percentile [66]. Karami et al. (2022) [88] found that the AA values ranged from 5.00 × 10^−4^ to 4.41 × 10^−1^ mg/kg. The study published by El-Zakhem et al. (2018) [89] is in line with the lowest value obtained (<5.00 × 10^−4^ mg/kg).

The OTA contamination in the samples analysed was 85%, with Peru and Mexico being the countries with the highest number of positive samples in instant coffee (100% in both). The high OTA percentages in instant coffee indicate that a greater effort must be made to control the presence of OTA to effectively safeguard human health. These results are lower than the 95% obtained by Vecchio et al. (2012) [59] and Yazdanfar et al. (2022) [90], and 100% by Aoyama et al. (2010) [91], Casal et al. (2014) [92], Galarce-Bustos et al. (2014) [93], T. P. Lee et al. (2012) [94] and Liu et al. (2008) [95]. In contrast, the values obtained in the present study are higher than the works of García-Moraleja et al. (2015) (33%) [96], Hajok et al. (2019) [97], Pokrzywa et al. (2022) [98] (43 and 63%, respectively) and Nielsen et al. (2015) (65%) [99].

The OTA concentrations we obtained were in the order of 1 × 10^−3^ mg/kg. Mexico and Brazil were the countries with the highest concentrations (1.71 × 10^−3^ mg/kg and 1.77 × 10^−3^ mg/kg, respectively). The ANOVA results indicated significant differences between the countries. It is noteworthy that the values obtained were below the limit allowed in the European Union (5.00 × 10^−3^ mg/kg) laid down in Commission Regulation (UE) 2023/915 [43]. These results are in agreement with those obtained by Galarce-Bustos et al. (2014) (1.80 × 10^−3^ mg/kg) [93], García-Moraleja et al. (2015) (4.93 × 10^−3^ mg/kg) [96], Hajok et al. (2019) (1.5 × 10^−3^ mg/kg) [97], Lee et al. (2012) (5.71 × 10^−3^ mg/kg) [94], Liu et al. (2008) (4.70 × 10^−3^ mg/kg) [95], Pokrzywa et al. (2022) (1.63 × 10^−3^ mg/kg) [98], Skarkova et al. (2013) (1.04 × 10^−3^ mg/kg) [100], Vecchio et al. (2012) (1.27 × 10^−3^ mg/kg) [59]. The studies by Aoyama et al. (2010), Casal et al. (2014) and Nielsen et al. (2015) stand out [91,92,99] with slightly lower values, in the order of 1E-04 mg/kg. Finally, values higher than 1 × 10^−2^ mg/kg and, therefore, above those permitted in the EU were obtained by Lindenmeier et al. (2011) [101] and Yazdanfar et al. (2022) [79]. According to a study by Gopinandhan et al. (2007) [102], high levels of OTA in instant coffee may be a consequence of adulteration with coffee husk, especially cherry husk. It is also known that when preparing instant coffee from blends with the robusta variety can directly lead to high levels of this mycotoxin [103,104,105]. Previous studies also concluded that OTA reduction by roasting ranges from zero to 100% [106,107]. Other studies focused on the stability of the toxin at processing temperatures and concluded that it is very stable [97,108]. The amount of OTA also depends on the season and storage conditions and time [97].

Table 3 shows the *pdf*-adjusted concentration of the hazards by country obtained using @Risk 8 software (Palisade, Newfield). For Cd and Hg, which were not detected, the concentration considered to characterise the risk was LOD/2 in both cases, i.e., 0.0025 mg/kg.

### 3.2. Risk Characterisation

**Figure 1:**
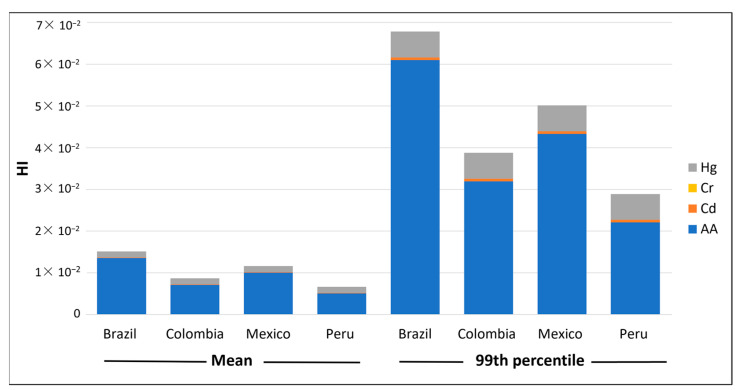
Mean and 99th percentile values of the HQ contribution of each chemical hazard to HI in different countries.

To assess both the non-genotoxic and genotoxic effects, we used metrics to characterise the risk from different countries (see Table 4). The HQ (for Cd, Cr, Hg and AA) and the HI values (summary of HQs) were used to estimate the partial and total chronic toxicity risk per country of Brazil, Colombia, Mexico and Peru, respectively (see Figure 1). As can be seen, all the findings were less than 1 in all cases, which indicates that the consumption of instant coffee, in relation to the non-genotoxic hazards studied, does not represent a level of concern.

The results indicated that AA (5.05 × 10^−3^ to 1.00 × 10^−2^) was the hazard with the highest HQ and, therefore, the major contributor to HI. The lowest HI value was found in Peru (6.63 × 10^−3^). Of the few previous works that characterised the risk in this product, Karami et al. (2022) [88] concluded that the HQ for AA determined in instant coffee purchased from different supermarkets in Tehran (Iran) was 2 × 10^−4^, indicating that the AA in instant coffee did not pose a health risk. Values lower than 1 were also found by Dybing et al. (2003) and Oroian et al. (2015) [109,110]. In the same vein, the calculated HQ for OTA in instant coffee was found to be below 1 [90,97], which indicated a safe level with no risk to human health.

**Figure 2:**
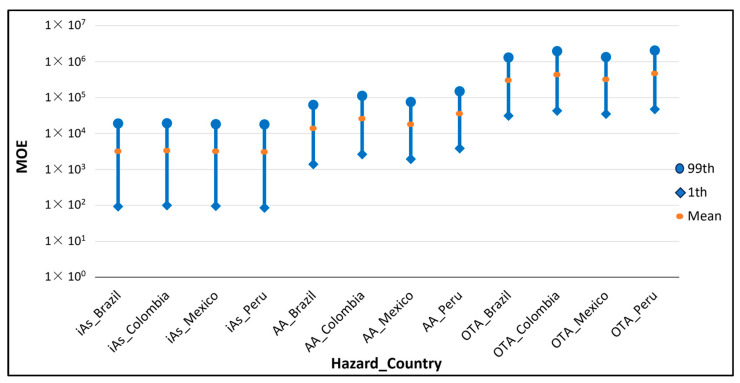
MOE for each country and hazard at the 1st, mean and 99th percentile when the BMDL considered was associated with a genotoxic effect (see Table 1).

Figure 2 shows the MOE distributions per country obtained for iAs, AA and OTA, considering the BMDL established for the genotoxic effects of these hazards. The results showed that the highest MOE was obtained in OTA, for which almost all the values of the country distributions were above the threshold of 10,000, making the level of concern acceptable, while all the distributions obtained for iAs were below the threshold (see Table 4), indicating that the risk should be considered of high concern. However, the Committee on Carcinogenicity of Chemicals in Food, Consumer Products and the Environment proposed that an MOE of 10 or higher, associated with a 0.5% increased risk of lung cancer in humans, for iAs could be considered of low concern [111]. According to the Swedish National Food Agency, a value between 1–10 should be considered a high to moderate risk, between 10–100 moderate to low, and higher values no risk [112]. Along the same lines, the results obtained from the POE metric, equal to zero, indicated that the exposure did not exceed the BMDL in any case and that the risk could be considered acceptable.

**Figure 3:**
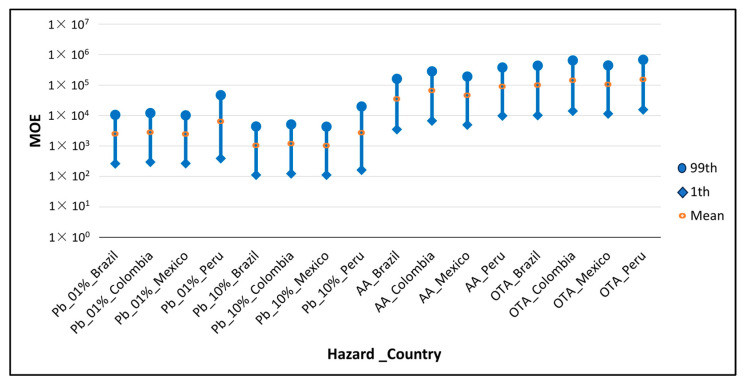
MOE for each country and hazard in the 1st, mean and 99th percentile, in which the BMDL considered was associated with cardiovascular, nephrotoxic and neurotoxic effects (see Table 1).

Figure 3 shows the MOE distributions obtained in the different countries for the non-genotoxic Pb effects (cardiovascular and nephrotoxic), AA and OTA. The findings show that AA ranged from 1 × 10^3^ to 1 × 10^5^ and OTA from 1 × 10^4^ to 1 × 10^6^, Table 4. Similar results were found by Karami et al., 2022, who obtained an MOE for the neurological effect of AA of 5 × 10^5^ and Yazdanfar et al., 2022, for the non-neoplastic effect of OTA of 2.78 × 10^3^ [88,90]. Pb had the smallest values, all being below the threshold of 10,000. However, the values were above 100, so taking into account the EFSA’s scientific opinion on lead in food, it was concluded that only when the MOE is <1 the possibility of effects on health cannot be excluded [65] and that the POE results were zero in all cases, which means that the exposure did not exceed the BMDL, indicating that the presence of all three hazards in instant coffee from the countries studied can be considered of low concern.

**Figure 4:**
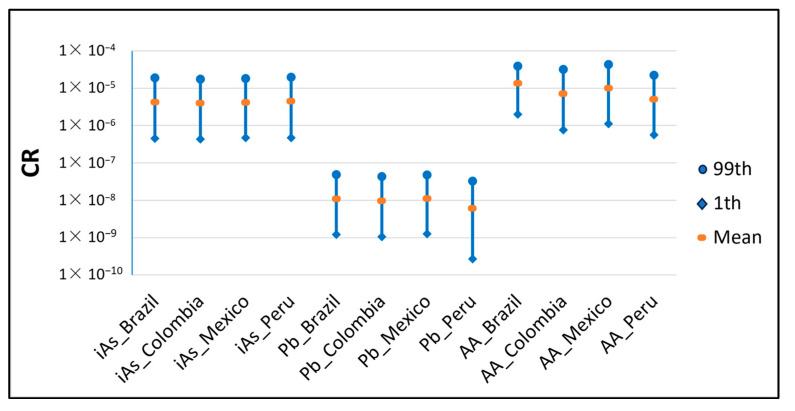
CR for each country and hazard at the 1st, mean and 99th percentile.

The CR or probability of an individual developing cancer over a lifetime due to the consumption of instant coffee was calculated for iAs, Pb and AA. Figure 4 represents the 1st percentile, mean and 99th percentile of the distribution obtained for each country and hazard. As can be seen, the results for Pb were lower than 1078 × 10^−6^, so this hazard in instant coffee does not result in any adverse health effects and the risk is considered low. However, almost the whole distribution is between 1.0 × 10^−4^ and 1.0 × 10^−6^ for the rest of the hazards, which is generally interpreted as a moderate risk.

## 4. Conclusions

The present study reports on the levels of iAs, Cd, Cr, Hg, Pb, AA and OTA in instant coffee manufactured in Brazil, Colombia, Mexico and Peru. The results showed that AA reached the highest concentrations and the differences per country were significant. In relation to risk characterisation, the non-genotoxic effect studied for Cd, Cr, Hg and AA based on HQ and HI metrics revealed that the consumers are within a safe range of risk. The MOE-POE combination calculated for the non-genotoxic effect of Pb, AA and OTA also indicated a low level of concern. Focusing on the genotoxic effect, iAs and AA obtained the lowest MOEs and the highest probabilities of cancer risk, indicating that these hazards involve a moderate risk. The results highlight the need to focus on the pre-production stages of the finished product in order to find appropriate and practical solutions to develop strategies that will mitigate the chemical risks in instant coffee. Attention should focus on issues such as the selection of the growing areas, fermentation conditions, enzymatic treatment of the raw material, alternative vacuum or steam roasting technologies, etc. Only in this way, and following the principle of the ALARA concept (as low as reasonably achievable) at each stage of the process, will it be possible to guarantee a high level of human health protection.

## Figures and Tables

**Table 2:** Metals above LOD, AA and OTA concentrations (mean, standard deviation (SD), minimum and maximum) in instant coffee from Brazil, Colombia, Mexico and Peru. ANOVA F-ratio per country.

Country of Origin		Concentration (mg/kg)				
		iAs	Cr	Pb	AA	OTA
Brazil (*n* = 72)	Mean	4.86 × 10^−2 a^	6.88 × 10^−3 a^	2.25 × 10^−2 a^	4.77 × 10^−1 c^	1.77 × 10^−3 b^
	SD	5.85 × 10^−3^	2.47 × 10^−3^	2.11 × 10^−3^	9.58 × 10^−2^	2.80 × 10^−4^
	Min	3.50 × 10^−2^	5.00 × 10^−3^	1.90 × 10^−2^	3.20 × 10^−1^	9.80 × 10^−4^
	Max	6.00 × 10^−2^	1.00 × 10^−2^	2.70 × 10^−2^	6.18 × 10^−1^	2.21 × 10^−3^
Colombia (*n* = 81)	Mean	4.68 × 10^−2 a^	6.67 × 10^−3 a^	2.00 × 10^−2 a^	2.50 × 10^−1 a,b^	1.26 × 10^−3 a^
	SD	6.22 × 10^−3^	2.40 × 10^−3^	2.90 × 10^−3^	3.60 × 10^−2^	2.80 × 10^−4^
	Min	3.50 × 10^−2^	5.00 × 10^−3^	1.70 × 10^−2^	1.87 × 10^−1^	3.20 × 10^−4^
	Max	5.40 × 10^−2^	1.00 × 10^−2^	2.50 × 10^−2^	3.05 × 10^−1^	1.64 × 10^−3^
Mexico (*n* = 12)	Mean	5.03 × 10^−2 a^	6.25 × 10^−3 a^	2.25 × 10^−2 a^	3.31 × 10^−1 b^	1.71 × 10^−3 b^
	SD	4.86 × 10^−3^	2.50 × 10^−3^	1.29 × 10^−3^	4.51 × 10^−2^	1.73 × 10^−4^
	Min	4.30 × 10^−2^	5.00 × 10^−3^	2.10 × 10^−2^	2.98 × 10^−1^	1.46 × 10^−3^
	Max	5.30 × 10^−2^	1.00 × 10^−2^	2.40 × 10^−2^	3.98 × 10^−1^	1.85 × 10^−3^
Peru (*n* = 15)	Mean	5.16 × 10^−2 a^	8.00 × 10^−3 a^	2.12 × 10^−2 a^	1.77 × 10^−1 a^	1.23 × 10^−3 a^
	SD	6.19 × 10^−3^	2.74 × 10^−3^	1.79 × 10^−3^	1.07 × 10^−2^	4.49 × 10^−4^
	Min	4.30 × 10^−2^	5.00 × 10^−3^	1.90 × 10^−2^	1.63 × 10^−1^	9.80 × 10^−4^
	Max	6.00 × 10^−2^	1.00 × 10^−2^	2.30 × 10^−2^	1.87 × 10^−1^	2.03 × 10^−3^
ANOVA F-ratio		1.209 ^n.s.^	0.493 ^n.s.^	4.74 ^n.s.^	59.22 ***	12.91 ***

Different letters per column indicate statistically different groups (Tukey test, *p* < 0.05), n.s.: Not significant; *** *p* < 0.001.

**Table 3:** Distribution of the hazards studied in instant coffee by country of origin (mg/kg) (see Figure S2).

Country	iAs	Cr	Pb	AA	OTA
Brazil	Logistic (0.039; 0.049; 0.058)	Exponential (0.005; 0.006; 0.009)	Extravalue (0.019; 0.022; 0.0265)	Uniform (0.307; 0.631)	Logitic (0.001; 0.002; 0.002)
Colombia	Triangular (0.036; 0.047; 0.053)	Exponential (0.005; 0.006; 0.010)	Exponential (0.017; 0.0189; 0.0258)	Uniform (0.183; 0.309)	Logistic (0.001; 0.001; 0.002)
Mexico	Uniform (0.043; 0.053)	Uniform (0.005; 0.01)	Uniform (0.021; 0.024)	Uniform (0.298; 0.398)	Uniform (0.001; 0.002)
Peru	Uniform (0.039; 0.064)	Uniform (0.004; 0.011)	Uniform (0.002; 0.023)	Uniform (0.157; 0.193)	Exponential (0.001; 0.001; 0.002)

**Table 4:** Risk characterisation per country using HQ, HI, MOE and CR metrics considering non-genotoxic and genotoxic effects.

Metrics	Hazard	Country of Origin	Mean	1st	5th	50th	95th	99th
HQ	Cd	All countries	1.44 × 10^−7^	1.62 × 10^−5^	2.24 × 10^−5^	1.04 × 10^−4^	4.00 × 10^−4^	6.22 × 10^−4^
	Cr	Brazil	2.61 × 10^−7^	2.58 × 10^−8^	3.78 × 10^−8^	1.80 × 10^−7^	7.60 × 10^−7^	1.24 × 10^−6^
		Colombia	2.54 × 10^−7^	2.59 × 10^−8^	3.71 × 10^−8^	1.76 × 10^−7^	7.31 × 10^−7^	1.19 × 10^−6^
		Mexico	2.88 × 10^−7^	2.90 × 10^−8^	4.29 × 10^−8^	2.02 × 10^−7^	8.24 × 10^−7^	1.29 × 10^−6^
		Peru	2.89 × 10^−7^	2.48 × 10^−8^	3.89 × 10^−8^	1.96 × 10^−7^	8.47 × 10^−7^	1.34 × 10^−6^
	Hg	All countries	1.44 × 10^−7^	1.62 × 10^−4^	2.24 × 10^−4^	1.04 × 10^−3^	4.00 × 10^−3^	6.22 × 10^−3^
	AA	Brazil	1.35 × 10^−2^	1.34 × 10^−3^	2.01 × 10^−3^	9.44 × 10^−3^	3.92 × 10^−2^	6.10 × 10^−2^
		Colombia	7.08 × 10^−3^	7.60 × 10^−4^	1.07 × 10^−3^	5.01 × 10^−3^	2.00 × 10^−2^	3.20 × 10^−2^
		Mexico	1.00 × 10^−2^	1.11 × 10^−3^	1.55 × 10^−3^	7.14 × 10^−3^	2.81 × 10^−2^	4.33 × 10^−2^
		Peru	5.05 × 10^−3^	5.67 × 10^−4^	7.84 × 10^−4^	3.61 × 10^−3^	1.41 × 10^−2^	2.21 × 10^−2^
HI	∑HQ	Brazil	1.51 × 10^−2^	1.52 × 10^−3^	2.24 × 10^−3^	1.05 × 10^−2^	4.30 × 10^−2^	7.07 × 10^−2^
	∑HQ	Colombia	8.67 × 10^−3^	9.10 × 10^−4^	1.32 × 10^−3^	6.13 × 10^−3^	2.48 × 10^−2^	3.76 × 10^−2^
	∑HQ	Mexico	1.16 × 10^−2^	1.27 × 10^−3^	1.78 × 10^−3^	8.27 × 10^−3^	3.27 × 10^−2^	5.10 × 10^−2^
	∑HQ	Peru	6.63 × 10^−3^	7.45 × 10^−4^	1.03 × 10^−3^	4.73 × 10^−3^	1.88 × 10^−2^	2.87 × 10^−2^
MOE	iAs	Brazil	3.18 × 10^3^	9.34 × 10	2.24 × 10^2^	1.78 × 10^3^	1.13 × 10^4^	1.91 × 10^4^
		Colombia	3.36 × 10^3^	1.00 × 10^2^	2.31 × 10^2^	1.90 × 10^3^	1.19 × 10^4^	1.94 × 10^4^
		Mexico	3.20 × 10^3^	9.66 × 10	2.24 × 10^2^	1.81 × 10^3^	1.13 × 10^4^	1.82 × 10^4^
		Peru	3.04 × 10^3^	8.55 × 10	2.10 × 10^2^	1.71 × 10^3^	1.08 × 10^4^	1.81 × 10^4^
	Pb *	Brazil	2.47 × 10^3^	2.63 × 10^2^	4.11 × 10^2^	1.62 × 10^3^	7.51 × 10^3^	1.05 × 10^4^
		Colombia	2.82 × 10^3^	2.94 × 10^2^	4.65 × 10^2^	1.85 × 10^3^	8.64 × 10^3^	1.21 × 10^4^
		Mexico	2.45 × 10^3^	2.67 × 10^2^	4.14 × 10^2^	1.61 × 10^3^	7.43 × 10^3^	1.02 × 10^4^
		Peru	6.41 × 10^3^	3.89 × 10^2^	6.52 × 10^2^	3.38 × 10^3^	2.22 × 10^4^	4.71 × 10^4^
	Pb **	Brazil	1.04 × 10^3^	1.11 × 10^2^	1.73 × 10^2^	6.81 × 10^2^	3.16 × 10^3^	4.41 × 10^3^
		Colombia	1.19 × 10^3^	1.24 × 10^2^	1.95 × 10^2^	7.78 × 10^2^	3.63 × 10^3^	5.09 × 10^3^
		Mexico	1.03 × 10^3^	1.12 × 10^2^	1.74 × 10^2^	6.78 × 10^2^	3.12 × 10^3^	4.27 × 10^3^
		Peru	2.69 × 10^3^	1.63 × 10^2^	2.74 × 10^2^	1.42 × 10^3^	9.33 × 10^3^	1.98 × 10^4^
	AA	Brazil	1.38 × 10^4^	1.39 × 10^3^	2.17 × 10^3^	9.00 × 10^3^	4.21 × 10^4^	6.31 × 10^4^
		Colombia	2.59 × 10^4^	2.66 × 10^3^	4.25 × 10^3^	1.69 × 10^4^	7.91 × 10^4^	1.12 × 10^5^
		Mexico	1.80 × 10^4^	1.96 × 10^3^	3.03 × 10^3^	1.19 × 10^4^	5.49 × 10^4^	7.60 × 10^4^
		Peru	3.57 × 10^4^	3.85 × 10^3^	5.99 × 10^3^	2.36 × 10^4^	1.08 × 10^5^	1.50 × 10^5^
	OTA	Brazil	3.03 × 10^5^	3.13 × 10^4^	4.96 × 10^4^	1.97 × 10^5^	9.17 × 10^5^	1.32 × 10^6^
		Colombia	4.34 × 10^5^	4.31 × 10^4^	6.83 × 10^4^	2.80 × 10^5^	1.32 × 10^6^	1.97 × 10^6^
		Mexico	3.23 × 10^5^	3.52 × 10^4^	5.38 × 10^4^	2.12 × 10^5^	9.79 × 10^5^	1.36 × 10^6^
		Peru	4.66 × 10^5^	4.78 × 10^4^	7.51 × 10^4^	3.01 × 10^5^	1.43 × 10^6^	2.06 × 10^6^
CR	iAs	Brazil	4.24 × 10^−6^	4.49 × 10^−7^	6.46 × 10^−7^	3.04 × 10^−6^	1.19 × 10^−5^	1.87 × 10^−5^
		Colombia	4.00 × 10^−6^	4.31 × 10^−7^	6.13 × 10^−7^	2.87 × 10^−6^	1.12 × 10^−5^	1.75 × 10^−5^
		Mexico	4.15 × 10^−6^	4.72 × 10^−7^	6.42 × 10^−7^	2.96 × 10^−6^	1.16 × 10^−5^	1.81 × 10^−5^
		Peru	4.46 × 10^−6^	4.68 × 10^−7^	6.73 × 10^−7^	3.14 × 10^−6^	1.27 × 10^−5^	1.97 × 10^−5^
	Pb	Brazil	1.10 × 10^−8^	1.21 × 10^−9^	1.70 × 10^−9^	7.87 × 10^−9^	3.10 × 10^−8^	4.83 × 10^−8^
		Colombia	9.74 × 10^−9^	1.05 × 10^−9^	1.47 × 10^−9^	6.88 × 10^−9^	2.74 × 10^−8^	4.33 × 10^−8^
		Mexico	1.10 × 10^−8^	1.25 × 10^−9^	1.71 × 10^−9^	7.90 × 10^−9^	3.08 × 10^−8^	4.76 × 10^−8^
		Peru	6.08 × 10^−9^	2.71 × 10^−10^	5.73 × 10^−10^	3.77 × 10^−9^	1.95 × 10^−8^	3.27 × 10^−8^
	AA	Brazil	1.35 × 10^−5^	1.34 × 10^−6^	2.01 × 10^−6^	9.44 × 10^−6^	3.92 × 10^−5^	6.10 × 10^−5^
		Colombia	7.08 × 10^−6^	7.60 × 10^−7^	1.07 × 10^−6^	5.01 × 10^−6^	2.00 × 10^−5^	3.20 × 10^−5^
		Mexico	1.00 × 10^−5^	1.11 × 10^−6^	1.55 × 10^−6^	7.14 × 10^−6^	2.81 × 10^−5^	4.33 × 10^−5^
		Peru	5.05 × 10^−6^	5.67 × 10^−7^	7.84 × 10^−7^	3.61 × 10^−6^	1.41 × 10^−5^	2.21 × 10^−5^

* MOE (BMDL01) = 1.50 × 10^−3^ mg/kg-Bw/day (Cardiovascular effect); ** MOE (BMDL10) = 6.30 × 10^−4^ mg/kg-Bw/day (Nephrotoxic effect).

## Data Availability

The original contributions presented in the study are included in the article and Supplementary Material, further inquiries can be directed to the corresponding authors.

## Supplementary Materials

The following supporting information can be downloaded at: https://www.mdpi.com/article/10.3390/foods13050726/s1; Figure S1: Comparison of the metal concentrations (iAs, Cd, Cr and Pb), AA and OTA in instant coffee found by other authors and those obtained in this study; Figure S2: Distributions for each hazard and country.
