# Supplementary material for: Probabilistic Risk Assessment of Metals, Acrylamide and Ochratoxin A in Instant Coffee from Brazil, Colombia, Mexico and Peru
Source: Foods. 2024 Feb 27;13(5):726. doi: 10.3390/foods13050726 (PMC10931501; doi:10.3390/foods13050726)
Supplement: Supplementary file 1 [file foods-13-00726-s001.zip › Figure S1.pdf]

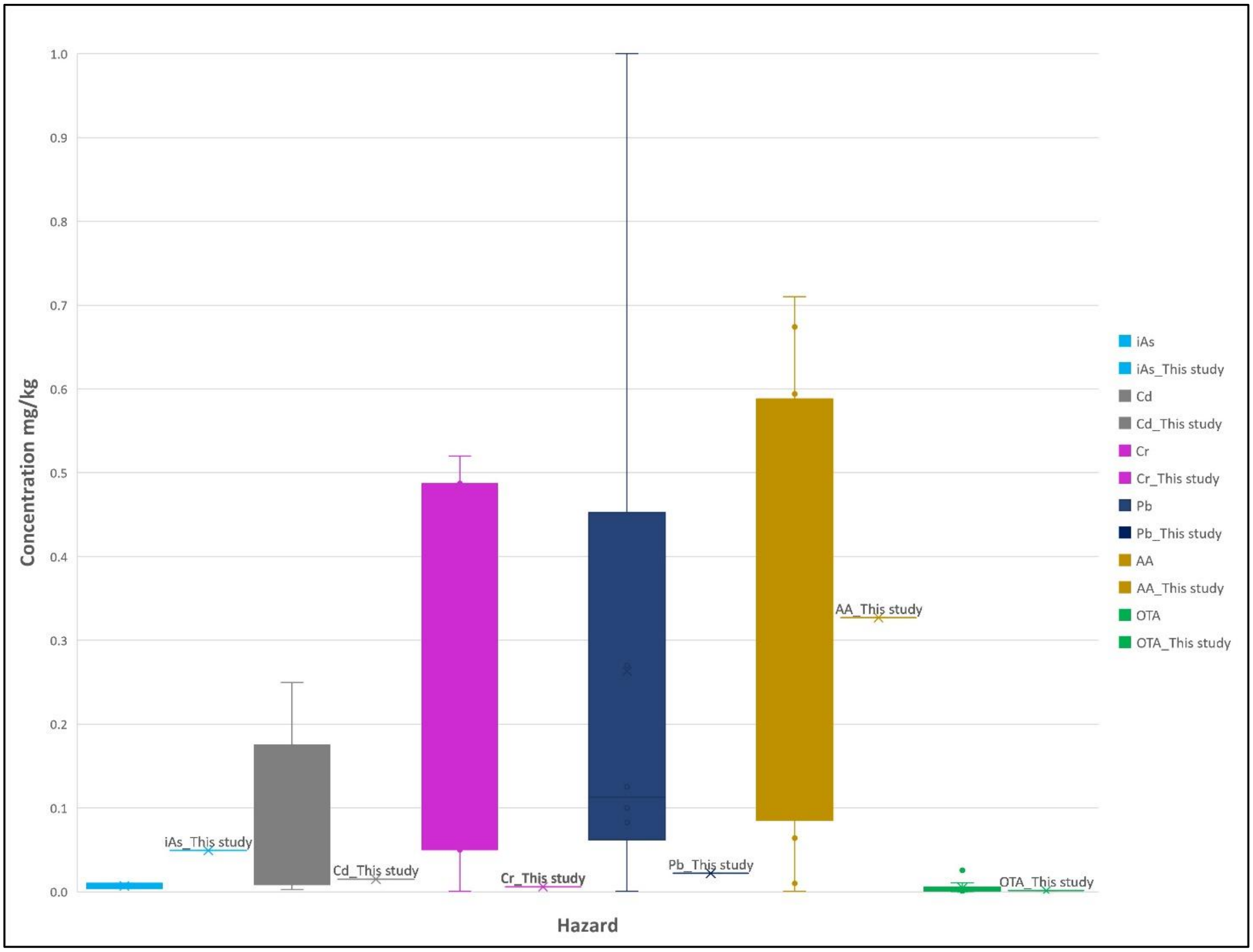

Figure S1. Comparison of the concentration of metals (iAs, Cd, Cr and Pb), AA and OTA in instant coffee found by other authors and the obtained in this study.
