# Supplementary figures and images for: Probabilistic Risk Assessment of Metals, Acrylamide and Ochratoxin A in Instant Coffee from Brazil, Colombia, Mexico and Peru
Source: Foods. 2024 Feb 27;13(5):726. doi: 10.3390/foods13050726 (PMC10931501; doi:10.3390/foods13050726)

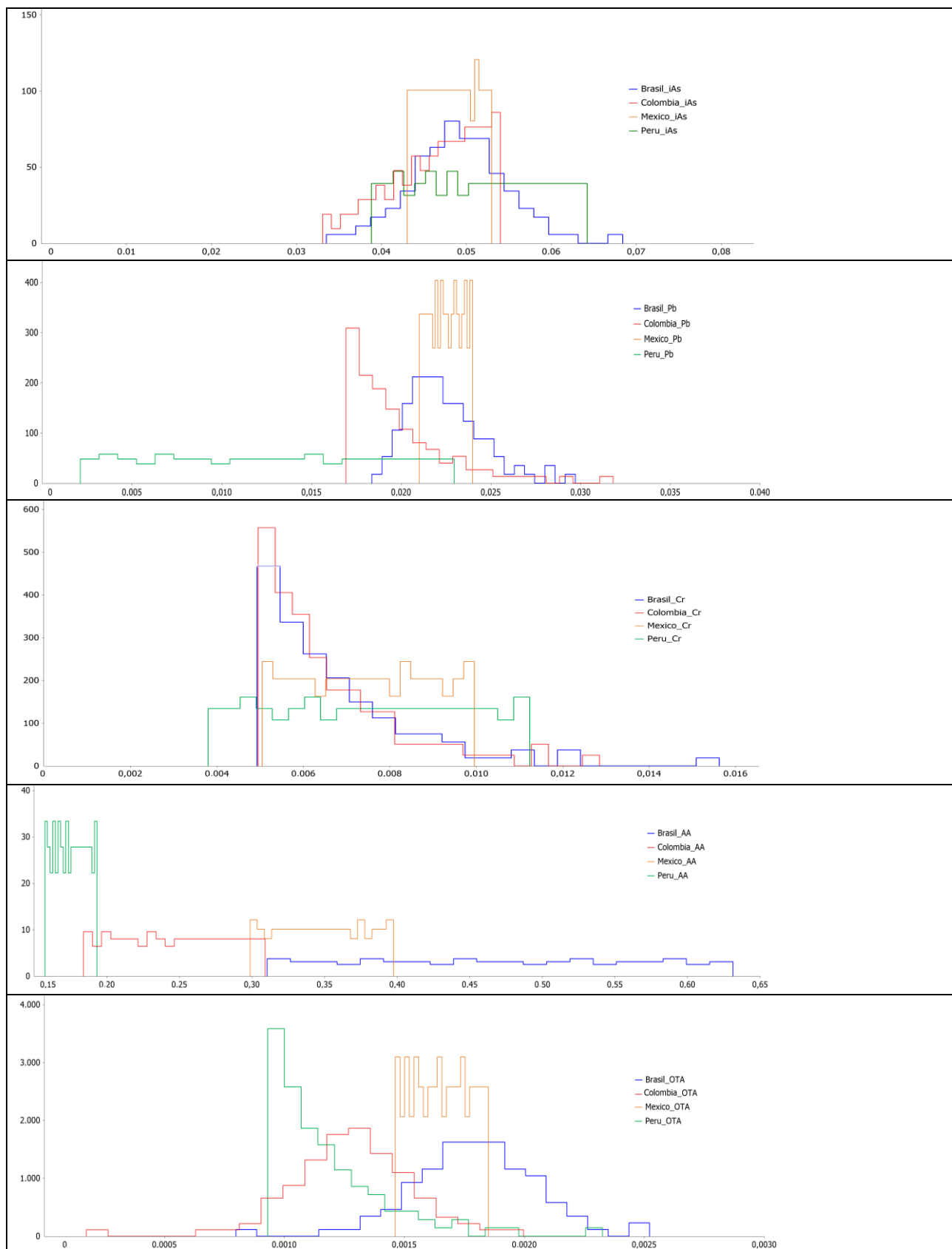

**Figure S2.** Distributions concentration for each hazard and country.

Supplement: Supplementary file 1 [file foods-13-00726-s001.zip › Figure S2.pdf]
